# Complete genome sequence of *Elizabethkingia* sp*.* GTC18459 isolated from a snake skin in Japan

**DOI:** 10.1128/mra.00119-26

**Published:** 2026-07-02

**Authors:** Masahiro Hayashi, Jun Yonetamari, Yoshinori Muto, Kaori Tanaka

**Affiliations:** 1 Institute for Glyco-core Research iGCORE, Gifu University 12785, Gifu City, Gifu, Japan; 2 Division of Anaerobe Research, Life Science Research Center, Gifu University 12785, Gifu City, Gifu, Japan; 3 Gifu University Center for Conservation of Microbial Genetic Resource, Gifu, Japan; 4 Division of Clinical Laboratory, Gifu University Hospital 476117, Gifu, Japan; 5 United Graduate School of Drug Discovery and Medical Information Sciences, Gifu University 12785, Gifu City, Gifu, Japan; Indiana University Bloomington, Bloomington, Indiana, USA

**Keywords:** *Elizabethkingia*, complete genome, pet animal

## Abstract

*Elizabethkingia* is a genus of bacterium in the order of *Flavobacteriales*. Here, we report the complete genome sequence of an *Elizabethkingia* species obtained from a snake skin in Japan. The genome comprised a circular chromosome of length 2,225,501 bp.

## ANNOUNCEMENT

Identifying pathogens associated with pet animals through genomic analysis is important for understanding their pathogenic potential and the zoonotic risks they pose (1, 2). Members of the genus *Elizabethkingia* are widely distributed in the environment and include opportunistic human pathogens (3, 4). Here, we report the genomic analysis of strain GTC18459, isolated in 2024 from the skin of a healthy *Python regius* kept at a pet shop in Seki City, Gifu, Japan.

The strain was cultured on tryptic soy agar with sheep blood at 28°C for 48 h under aerobic conditions and purified twice. Colonies were suspended in TE buffer and pelleted by centrifugation. Genomic DNA was extracted using the NucleoBond HMW DNA Kit (Macherey-Nagel, Japan). For identification, the 16S rRNA gene was amplified and sequenced by Sanger sequencing using an ABI 3500xl DNA Analyzer (Thermo Fisher Scientific, Waltham, MA, USA). The sequence was compared with those in the NCBI nucleotide database using BLASTn via the NCBI web server. BLASTn analysis (5) indicated that the strain belonged to the genus *Elizabethkingia*, showing the highest similarity to *Elizabethkingia meningoseptica* ATCC 13253 (type strain accession no. AJ704540) with 93.5% similarity.

Whole-genome sequencing was performed using PromethION 2i (Oxford Nanopore Technologies) and DNBSEQ-G400 (MGI Tech) for long-read and short-read sequencing, respectively (6–8). A library was constructed with a Native Barcoding Kit (SQK-NBD114-24; ONT) without shearing for long-read sequencing. PromethION 2i (ONT) with a FLO-PRO114M flow cell was used. Dorado v.7.2.14 was used for base calling, and raw reads were trimmed and quality-filtered using NanoFilt v.2.7.1 (9). MGIEasy FS DNA Library Prep Set (MGI Tech) was used to construct a “short-read” library, and 2 × 150 bp paired-end sequencing was performed on the DNBSEQ-G400 platform. Read quality was assessed using fastp v.0.20.1 and NanoPlot v.1.32.1 (10). The quality for short reads showed over 95.4% of bases >Q30, while long reads demonstrated a mean quality score of 13.7. Genome assembly was performed using Flye v.2.9 with default parameters (11), followed by polishing with both reads. Assembly circularization was automatically detected by Flye, and the assembly was rotated to begin with *dnaA* on the forward strand. The contig graph was visualized using Bandage v.0.8.1 (12), and taxonomic integrity was evaluated using Blobtools v.1.0 with short reads (13).

Table 1 summarizes the genomic information. Genome statistics and quality were assessed using SeqKit (v.2.9.0) (14) and CheckM (v.1.2.2) with the lineage workflow (15), which confirmed 100% genome completeness with 0% contamination. Genome annotation using the DDBJ Fast Annotation and Submission Tool v.1.2.0 (16) predicted 2,035 coding sequences, 9 rRNA genes, 46 tRNA genes, and 2 CRISPR sequences. Species identification based on average nucleotide identity using GTDB-Tk v.2.3.2 was inconclusive (17). Taxonomic analysis using the Type Strain Genome Server (TYGS) identified *E. meningoseptica* as the closest species; however, the digital DNA–DNA hybridization value between GTC18459 and the *E. meningoseptica* type strain genome was 18.3%, which was well below the species delineation threshold of 70% (18, 19).

**TABLE 1:** Information on the complete genome sequence of an *Elizabethkingia* sp. strain isolated from a snake skin in Japan

Parameter	Strain name
	*Elizabethkingia* sp. GTC18459
DNBSEQ sequencing^*a*^	
No. of reads	4,816,688
Size (kb)	722,503
Avg coverage (×)	324.6
DRA accession no.	DRR888609
ONT sequencing^*a*^	
No. of reads	110,943
Size (kb)	443,707
Avg read length (bp)	43,997
Avg coverage (×)	199.4
N50	44,961
DRA accession no.	DRR888608
Assembly	
Assembly N50 (bp)^*c*^	2,225,501
Estimated genome completeness (%)^*c*^	100.0
Estimated genome contamination (%)^*c*^	0.0
Genome structure	One chromosome
DDBJ/GenBank accession no.	AP045222
Genome size (bp)	2,225,501
GC content (%)	47.8
No. of coding sequences^*b*^	2,035
Number of rRNAs^*b*^	9
Number of tRNAs^*b*^	46
Number of CRISPRs^*b*^	2

^*a*^ DRA, DDBJ Sequence Read Archive.

^*b*^ DFAST, DDBJ Fast Annotation and Submission Tool.

^*c*^ Determined with SeqKit v.2.9.0.

## Data Availability

The genome sequence of *Elizabethkingia* sp. GTC18459 has been deposited in DDBJ under accession number AP045222. Raw reads are available in the DDBJ Sequence Read Archive under accession numbers DRR888608 and DRR888609.
